# Lessons learned from recruiting into a longitudinal remote measurement study in major depressive disorder

**DOI:** 10.1038/s41746-022-00680-z

**Published:** 2022-09-03

**Authors:** Carolin Oetzmann, Katie M. White, Alina Ivan, Jessica Julie, Daniel Leightley, Grace Lavelle, Femke Lamers, Sara Siddi, Peter Annas, Sara Arranz Garcia, Josep Maria Haro, David C. Mohr, Brenda W. J. H. Penninx, Sara K. Simblett, Til Wykes, Vaibhav A. Narayan, Matthew Hotopf, Faith Matcham, RADAR-CNS consortium

**Affiliations:** 1 Institute of Psychiatry, Psychology and Neuroscience, King’s College London, London, UK; 2 South London and Maudsley NHS Foundation Trust, London, UK; 3 Academic Department of Military Mental Health, King’s College London, London, UK; 4 Department of Psychiatry and Amsterdam Public Health Research Institute, Amsterdam UMC, Vrije Universiteit, Amsterdam, The Netherlands; 5 Parc Sanitari Sant Joan de Déu, Fundació Sant Joan de Déu, CIBERSAM, Universitat de Barcelona, Barcelona, Spain; 6 H. Lundbeck A/S, Valby, Denmark; 7 Center for Behavioral Intervention Technologies, Department of Preventative Medicine, Northwestern University, Chicago, IL USA; 8 Janssen Research and Development, LLC, Titusville, NJ USA; 9 School of Psychology, University of Sussex, Falmer, East Sussex UK

**Keywords:** Depression, Clinical trial design

## Abstract

The use of remote measurement technologies (RMTs) across mobile health (mHealth) studies is becoming popular, given their potential for providing rich data on symptom change and indicators of future state in recurrent conditions such as major depressive disorder (MDD). Understanding recruitment into RMT research is fundamental for improving historically small sample sizes, reducing loss of statistical power, and ultimately producing results worthy of clinical implementation. There is a need for the standardisation of best practices for successful recruitment into RMT research. The current paper reviews lessons learned from recruitment into the Remote Assessment of Disease and Relapse- Major Depressive Disorder (RADAR-MDD) study, a large-scale, multi-site prospective cohort study using RMT to explore the clinical course of people with depression across the UK, the Netherlands, and Spain. More specifically, the paper reflects on key experiences from the UK site and consolidates these into four key recruitment strategies, alongside a review of barriers to recruitment. Finally, the strategies and barriers outlined are combined into a model of lessons learned. This work provides a foundation for future RMT study design, recruitment and evaluation.

## Introduction

The use of mobile technology in healthcare (mobile health; mHealth) has the potential to revolutionise both clinical practice and research^1^. Novel remote measurement technologies (RMTs), for example, smartphone applications, sensors and wearable technologies, are a subsection of mHealth, and can enable frequent, longitudinal and personalised health monitoring^2^. Currently, the assessment, and subsequent treatment, of many chronic health conditions is limited to retrospective recall during routine clinic visits, which can be biased by cognitive and memory heuristics^3^, and social desirability bias^4^. RMT data offers the potential for high-frequency symptom monitoring, which is more reflective of an individual’s daily experience^5^.

RMT data hold particular relevance for conditions of a recurrent nature. Major depressive disorder (MDD) is a mental disorder characterised by persistent low mood or anhedonia, often fluctuating between periods of remission and recurrence. The prevalence of MDD worldwide increased 18.4% from 2005 to 2015^6^. The economic burden of MDD is currently estimated at $326 billion (2020 values), exacerbated further by an increased risk of comorbidities and healthcare resource utilisation in those with high relapse and recurrence rates^7,8^. Data obtained through a combination of smartphone questionnaires (active RMT; aRMT) and inbuilt sensors (passive RMT; pRMT) from phones and wearables can provide rich, multi-parametric information on symptom change, risk factors, cognition, sleep and diurnal patterns of behaviour, sociability and physical activity^2^. Crucially, the integration of RMTs into MDD research could provide the temporal resolution needed to detect indicators of future depressive episodes^9^.

For the potential of RMTs to be achieved, it is important to understand whether and how people engage with research studies of this nature. Engagement is a multi-stage construct indicating the extent to which a resource is actively used^10^. Examining engagement with the research protocol in RMT studies, for example via recruitment rates, provides a necessary first step^11^. Whilst findings suggest that people with MDD endorse the view that RMTs could be used to detect and predict relapses^12,13^, work has highlighted potential barriers to recruitment into such research. These include user-related factors, such as personal agency and privacy concerns, as well as system-related factors, including perceived ease of use and convenience^12,14^. In practice, RMT studies currently present small sample sizes^15^; a recent systematic review of cross-sectional, case-control, cohort and RCT studies found the median sample size of the 51 identified studies to be *N* = 58 (ranging from *N* = 6 to *N* = 1714)^16^. Recruitment rates into mental health trials range between 0 and 75%^17^. Reduced help-seeking and motivational behaviours are often cited as reasons for low engagement with research on depression^18,19^. Low recruitment rates present several limitations, including an increased risk of selection bias, a loss of statistical power and reduced generalisability, hindering the ability to produce valid results worthy of implementation into clinical practice^20^. Despite calls for adherence to Strengthening the Reporting of Observational Studies in Epidemiology (STROBE^21^) guidelines, recruitment strategies and non-participation rates are not reported in the majority of RMT studies^16^. Thus, little is currently known about the ways in which recruitment can be maximised in research of this nature.

The need to understand recruitment into remote measurement studies has increased exponentially since the COVID-19 pandemic. Isolation and social distancing have forced both clinical research and practice to halt face-to-face appointments. Healthcare services are rapidly seeking innovative ways to monitor health remotely, not least for contact tracing of COVID-19 symptoms, but also for continued measurement of pre-existing conditions outside of the clinic^22^. Recruitment into studies of smartphone apps designed for tracking COVID-19 symptoms shows initial promise, reflecting a national effort to combat the pandemic^23,24^. A fundamental challenge in healthcare will be coordinating a sustained response beyond these times, during which adoption of, and engagement with, remote (mental) health tracking has been noted as of great importance^25–27^.

Currently, a framework of best practice for recruiting into RMT studies does not exist. In the wider mHealth literature, an emerging trend for papers sharing recruitment experiences has encouraged standardisation and evaluation in the field^28^, spanning common mental health conditions^29^, cardiovascular health^30^ and risky health behaviours^28^. However, RMTs fundamentally differ from mHealth interventions in that they require sustained engagement with observational data collection over long periods, crucially offering no tangible or immediate benefits to the user. Druce et al.^31^ reviewed successful recruitment into two aRMT apps for symptom tracking in rheumatoid arthritis between 30 days and 12 months. However, there is no clear consensus as to whether these considerations can be applied to multi-parametric studies that track depression for a longer time period, given the need to offset extra participant burden or address additional privacy concerns. Efforts to translate this body of work into the field of multi-parametric RMT research could provide the necessary first steps in understanding how best to recruit into such studies, and in doing so, produce high-quality results across academia and beyond.

### The RADAR-MDD study

The Remote Assessment of Disease and Relapse-Major Depressive Disorder (RADAR-MDD) study is the largest multi-parametric, multi-site, longitudinal prospective cohort study to date (as part of the wider RADAR-Central Nervous System (CNS) public-private partnership study: https://www.radar-cns.org/). The study utilised the RADAR-base system^32^ to collect a wealth of data from aRMT (a smartphone app delivering validated mood questionnaires, cognitive games, speech tasks and an electronic diary) and pRMT (a wearable fitness device measuring ambient noise and light, Bluetooth connections, GPS locations) sources to predict depressive relapse (the primary outcome measure). Further information is available in the protocol paper^33^. Eligible participants met with the research team for a single enrolment session, before being followed up remotely for a minimum of 6 months, a maximum of 3 years. The study obtained ethical approval from the Camberwell St Giles Research Ethics Committee (REC reference: 17/LO/1154) in London, from the CEIC Fundacio Sant Joan de Deu (CI: PIC-128–17) in Barcelona, and from the Medische Ethische Toetsingscommissie VUms (METc VUmc registratienummer: 2018.012 – NL63557.029.17) in the Netherlands. All participants provided written informed consent.

RADAR-MDD aimed to recruit 600 participants across the UK, Spain, and the Netherlands. Recruitment began in November 2017 at the London, UK site, acting as a year-long pilot for the recruitment and enrolment process before the remaining two sites joined in September 2018 and February 2019 respectively. By end of follow-up in June 2020, RADAR-MDD had exceeded its overall recruitment target (*N* = 623), and in the London site alone had recruited 175% (*N* = 350) of its target sample, making it the largest longitudinal observational cohort study of RMTs to date^2,16^. It was also able to sustain a steady level of recruitment throughout the COVID-19 pandemic. Thus, the study provides an opportunity to increase the transparency of reporting on recruitment and common barriers for non-participation in this field, as a multi-parametric RMT study with an extensive follow-up period.

The current paper aims to reflect on the experiences of recruiting into the RADAR-MDD project’s UK site. Here the UK site was chosen as many of these experiences were collated as part of the year-long pilot study for the recruitment and enrolment procedures; learnings that were then implemented at the other sites when these joined. The current findings were collected over the entirety of the study in discussions with researchers across all three sites, the patient advisory board, direct participant feedback during the procedures, and informal focus groups with other key stakeholders.

More specifically, this paper will present key reflections on successful recruitment strategies. It will then explore some main obstacles to recruitment, before merging findings into a model to reflect our main lessons learned. This work provides a starting point for the evaluation of recruitment pathways in RMT research, alongside suggestions for future work in the public or private sector to build upon.

## Reflections on recruitment strategies

The recruitment strategies that we reflect on from RADAR-MDD are split into four main areas of consideration. These key areas are: (1) the need for co-design with service-user involvement, (2) a recruitment team fulfilling key competencies, (3) minimising participant burden via streamlined procedures and (4) making enrolment accessible for all while being open to iterative change.

### Co-design with service-user involvement

Co-design with service users underpinned every aspect of the RADAR-MDD study design. User involvement was essential to ensure usability and interest in the technology and was especially relevant for studies that struggle with low uptake and adherence^17,34^. Users can reference personal lived experience when evaluating a system, and can provide valuable feedback on the design, enrolment procedures, communication materials, and implementation into clinical practice^35,36^.

RADAR-MDD included a work-package dedicated to Patient and Public Involvement (PPI). A series of work has been published, including systematic reviews and focus groups, with those with lived experience of MDD across three countries, exploring RMTs to manage depression^12,15^. This work-package also facilitated a Patient Advisory Board (PAB^33^). The PAB had input from the initial design, with service users participating in wearable device selection that culminated in using the Fitbit Charge device for data collection^13^, reviewing of participant-facing documents and app usability testing. In addition to the PAB, the UK site also consulted two local service user advisory groups at King’s College London regarding payment plans and feedback data. Service user involvement allowed for tailored procedures and materials, the most acceptable to the target population, which in turn minimised the burden of an observational longitudinal study and incentivised recruitment.

This person-based approach has been widely recommended in interventional designs^34^; we suggest that it is equally important in observational research. In utilising the expertise of the PAB, the RADAR-MDD study was designed for, and by, individuals with similar lived experiences to those being recruited. This created a smooth transition from protocol design to recruitment.

### The recruitment team

Through consultation with the PAB, and previous literature that cites human contact as a predictor of retention in longitudinal studies^20,37^, it became clear that a small participant-facing research team was essential. For the RADAR-MDD’s London site, this consisted of three full-time research assistants, who provided technological support and clinical risk assessments to participants. Here recruiting the right team, with the necessary competencies and providing training relevant to RMT study design, was essential.

Firstly, when hiring the patient-facing team, it was critical to consider the key competencies needed for an RMT study which features complexity in the study design, the technology used, and the likely support needed. In a systematic review of longitudinal clinical studies, eligible research team staff were screened for: specialist experience working with the target population, cultural competencies, communication skills, empathy and sensitivity^37^. For RMT studies specifically, we learned that patience and awareness of potential technological barriers is integral, as much of the contact with participants during recruitment involves communicating with individuals of varying technological abilities. The RADAR-MDD study London site screened for these competencies in behavioural interviews, consisting of a mock recruitment session with a colleague outside of the research team. The applicant was provided little information about the study in advance and asked to respond to questions from a mock participant. The aim of this activity was to assess situational judgement, and the ability to build social rapport, field participant questions, and create an atmosphere conducive to requesting further support if needed. This was based on work by Salado et al.^38^ indicating that conventional and behavioural interviews assess different constructs with the latter being more suited for this role.

During the recruitment period, it became clear that the team could benefit from additional staff to accommodate participant interest. To this end, a mental health nurse, based at the local NHS Foundation, was seconded to the team on a research placement for 12 months part-time. The benefits of this were three-fold: (1) increased capacity for recruitment calls and enrolment sessions; (2) provision of expert clinical advice for risk assessments; and (3) an attempt to accommodate participant queries on the use of RMTs in clinical practice.

Secondly, it was vital for recruitment staff to be trained on how to introduce the study most appropriately to the target population. Courteous and clear communication was key here, particularly when recruiting for an RMT study that featured complex designs and procedures for the participants. We learned that it is critical for staff to be able to discuss the study goals and procedures in a vernacular appropriate to the population, while following a standardised script. This was part of a larger training manual that was provided to the researchers, standardising the process across sites. Mental health cohorts may pose additional considerations, such as phone call anxiety, so the team needed to be confident in communicating procedures and diagnostic interviewing via various channels.

It was important for the research team to manage participant expectations by clearly explaining the participant burden and the aims of the study. This was particularly relevant as the RMT was not used as an intervention but rather as a data collection tool alone. Similarly, it was imperative that research staff could answer potential questions regarding technological concerns, data protection, data security, and confidentiality, as these present key areas of participant concern in RMT studies. This is especially relevant for longitudinal RMT studies, as participants are signing up to engage with (often new) technologies that will become a new part of their daily routine. This can seem daunting, thus a research team that can provide information that might eradicate concerns, and that is easily contactable throughout participation is likely to promote confidence in enroling. This highlights the importance of human contact during recruitment, regardless of the remote potential of the technologies. Using RMT in research is relatively novel, so the promise of a personable, knowledgeable, and trained research team, both during recruitment and throughout the study, was paramount to the success of study recruitment.

### Minimising participant burden

A third consideration for successful recruitment was a focus on reducing participant burden. Where RMT, in particular pRMT, is by design often ‘invisible’, the addition of a wide range of exploratory aRMT variables might cause individuals to feel overwhelmed by the amount of data that is being tracked, and the time and energy it will cost to incorporate the technology into their life. We reflect on two ways the RADAR-MDD study attempted to make the recruitment process as streamlined and rewarding as possible: (1) easy participant onboarding and (2) incentivisation for participation.

**Fig. 1:**
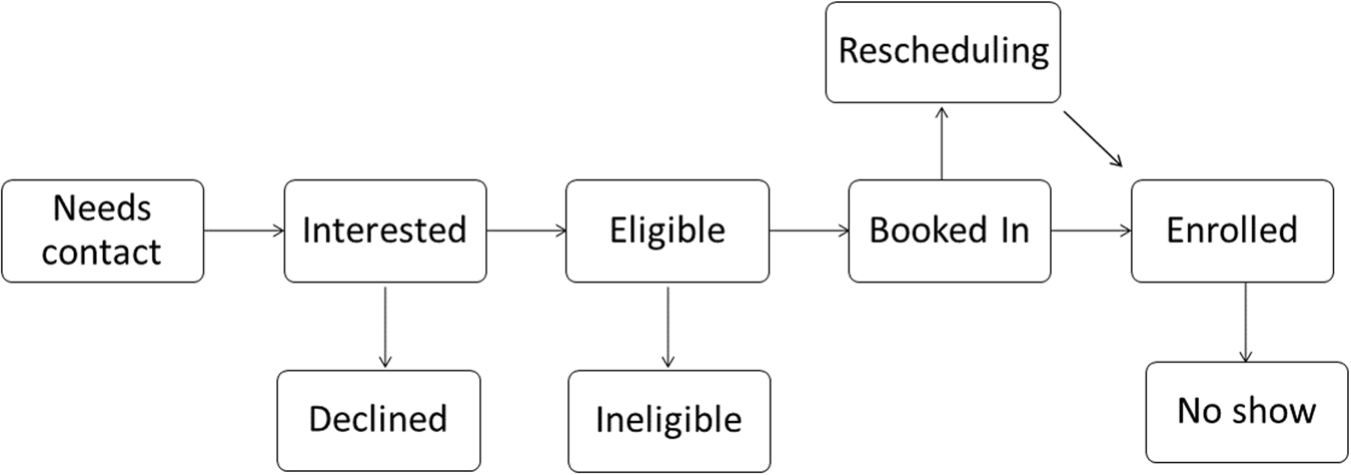
A process diagram of the various statuses from initial contact to enrolment. Participants progress from ‘needs contact’ (left) to ‘enrolled’ (right). Boxes along the bottom row detail non-participation statuses.

To streamline onboarding, the RADAR-MDD study created a standardised pathway to guide participants from initial contact to enrolment. This comprised of a system of statuses that helped identify, contact and track potential participant journeys to enrolment. The team used several methods to identify a pool of potentially eligible participants which included both team-initiated (Consent for Contact databases; C4C) and participant-initiated (form on the RADAR-CNS website) approaches. A central spreadsheet kept a log of contacts, whereby each individual was assigned a status from “needs contact” to “enrolled” (Fig. 1). This enabled the team to identify individuals quickly and progress them accordingly. Not only did this streamline the recruitment process, but it allowed for the easy creation of meaningful graphics to quantify the process to the extended team.

Another critical factor in offsetting participant burden was incentivisation. The debate over financial incentives for participants is a long-standing balancing act for ethics committees and principal investigators, regarding the influence over study design^39,40^. Commonly, agreement is found that allows for financial incentives when the risk to the participant is negligible and can result in more robust research outcomes^41^. When reflecting on the RADAR-MDD project, at the London site participants were paid £15 for the initial enrolment session and were reimbursed travel expenses. They were also informed that they would receive £5 for every 3-month outcome assessment they completed (long-form questionnaires sent via email) during the follow-up period. Additional, financial incentives were not offered for completion of aRMT questionnaires. This was based on feedback sessions with the RADAR-CNS PAB, the FAST-R group and research conducted by Mohr et al.^42^. Mohr et al. found that financial rewards can provide motivation for tasks that take more time, while these same rewards for engaging and quick tasks can be perceived as controlling or indicators that the individual lacks motivation.

In addition to monetary incentivisation, the offer of technology can be an intriguing prospect for potential participants. Here it was critical to provide equal opportunity and to address digital divide, as a substantial proportion of the population do not have access to smartphones and this group may be particularly relevant for mental health studies^43^. Thus, the RADAR-MDD study provided all technology free of charge (a Fitbit Charge device and an Android smartphone where not already owned). This was coupled with a technology user guide, and the offer of help with switching devices if required. As a result, owning or having experience with the technology was not a prerequisite for participation. This allowed for the recruitment of a diverse range of participants, some of whom had never used a smartphone before.

A third incentive for enrolment into RMT studies is moral alignment with the aims of the research, and the altruistic belief that engaging with such research will provide clinically valuable results^44^. During the RADAR-MDD recruitment process, participants were reminded at various points that the ultimate aims of the study were to implement findings into clinical practice. They were also informed that they could view some aspects of their data, during participation (e.g., heart rate, step counts on the FitBit dashboard) and as an infographic at the end (e.g., mood fluctuations across time)^33^. As a result, potential participants were encouraged to feel they were contributing to a tangible, innovative way of using technology to manage health, which will hopefully prove beneficial to themselves and the wider MDD community. Overall, we believe that combining a streamlined recruitment process with tailored incentives allowed for a reduction in participant burden and encouraged initial participation.

### Iterative process

Finally, we aimed to strike a delicate balance between protocol adherence for increased reliability, and the need for flexibility, for example in accommodating participant needs or incorporating feedback.

When designing enrolment procedures, it was imperative for the RADAR-MDD study to take a flexible approach that was malleable to differing levels of technological expertise and various personal circumstances (e.g., mental health affecting the ability to travel for enrolment). At the London site, RADAR-MDD participants were offered a choice of three enrolment formats: (1) research centre visit for in-person session, (2) a home visit, or (3) a video call (and a separate procedure that was accessible to those with hearing impairments). During the in-person and home visit sessions, a member of the research team would introduce the study, remind the participant of the aims and set up the technology for them. Online sessions followed a similar procedure, but the participant set up the devices themselves once received in the post, following live instructions. These options of different enrolment procedures provided participants the flexibility to make study enrolment as effortless and comfortable as possible.

To standardise enrolment procedures, the research team created scripts and checklists for each method. However, it became apparent from early participant feedback that certain aspects of the session could be adapted further to promote engagement. For example, early participants noted feeling uncomfortable wearing the Fitbit at night, leading researchers to advise to ‘wear the device as much as you feel comfortable’ during the enrolment sessions. Incorporating early feedback into the recruitment and enrolment protocol served to enhance clarity, particularly as RMT research is relatively novel.

As well as adapting to individuals, RMT study protocols lend themselves to changing environmental circumstances. Though unprecedented, the COVID-19 pandemic provided a case study for one of the key benefits of working with RMTs: structures are likely in place to continue with data collection and recruitment, during times when face-to-face contact is not permitted. The RADAR-MDD study was able to continue the onboarding process with relative ease during the UK lockdown. In part, this is reflective of the decision to re-contact individuals who were previously unreachable. However, it is also reflective of the little adaptation needed to switch to fully remote onboarding procedures, given that this was already an established method of enrolment. Online recruitment procedures (explained previously) were followed as usual, with the team contacting individuals via work mobiles from home office. Technology delivery through postal networks was also unaffected. Overall, the RADAR-MDD study was able to take advantage of the remote nature that these technologies afford and continue recruitment.

## Barriers to participation

**Fig. 2:**
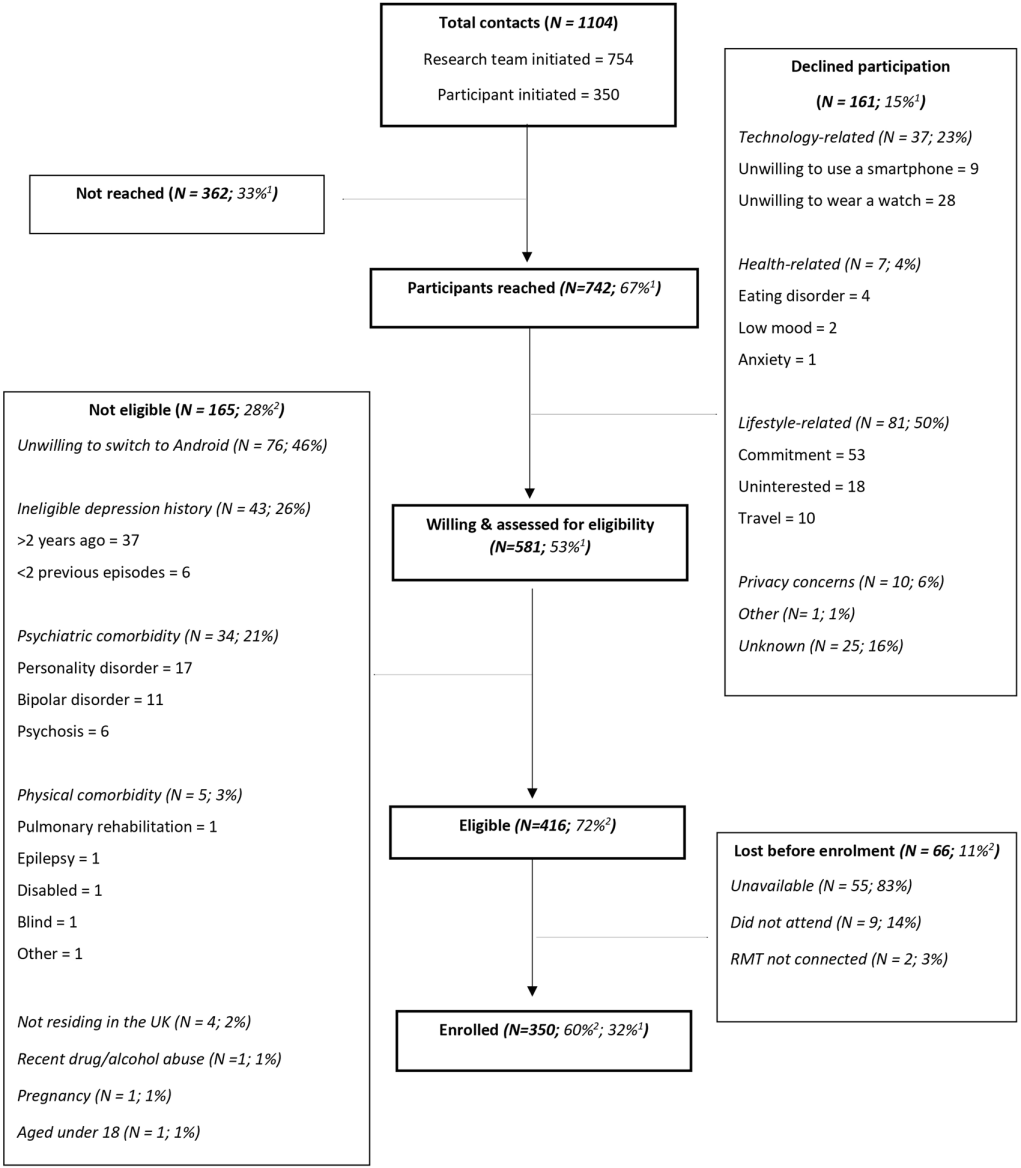
A recruitment flowchart from initial contact to enrolment for the RADAR-MDD London site. ^1^Of total contacts, denominator = 1104. ^2^Of willing & assessed for eligibility, denominator = 581.

When summarising our lessons learned in recruiting into a large-scale RMT study, it is also important to consider barriers to participation. In RADAR-MDD two key obstacles to recruitment were encountered: (1) technological barriers and (2) health-related barriers. Figure 2 shows the breakdown of reasons given for declining to participate in the study (*n* = 161) and ineligibility (*n* = 165). Here, we reflect on these and consider how we might have mitigated them. These data were collected during participant eligibility calls and logged as per the streamlined enrolment procedure outlined previously.

### Technology-related barriers

When considering barriers to enrolment for the RADAR-MDD study, participants’ unwillingness to switch to an Android device was the most common reason for ineligibility (46%). Here participants expressed having set up their homes/work on devices on alternative operating systems, e.g., iOS, making the switch impractical. This highlights the importance of RMT device compatibility with a wide range of operating systems. Meanwhile, 23% declined the use of a smartphone, or felt generally uncomfortable wearing a watch or jewellery on their wrists for long periods.

It is critical to reflect on and acknowledge the biases introduced by this barrier. For example, in the RADAR-MDD study, despite the provision of technological support, the sample could be biased towards people who are already using smart watches and smartphones. However, it is possible that this is reflective of the type of sample that would choose to use symptom tracking routinely should it be implemented in clinical practice.

### Health-related barriers

Another obstacle faced in RADAR-MDD recruitment was health-related barriers. An unexpected finding here was the necessity to reflect on the potential health impact the technology may have on certain comorbidities. For example, the presence of comorbid eating disorders made some (3%) decline participation. This was not a factor the team considered prior to recruitment, yet it highlights the importance of understanding the triggers that viewing some aspects of personal health data, in particular in relation to physical activity, might cause. Here, the potential effects of symptom monitoring on those with a history of eating disorders might be detrimental and thus had to be considered.

Of the reasons given for declining participation in the study, low mood only accounted for 1%. This was particularly interesting as it suggests that the presence of depression did not deter many individuals from participating, and indeed 61% of participants did report a current depressive episode at baseline. Conversely, it could be argued that the presence of low mood could prompt an interest in mood tracking, and thus an interest in participating.

## A model of lessons learned

**Fig. 3:**
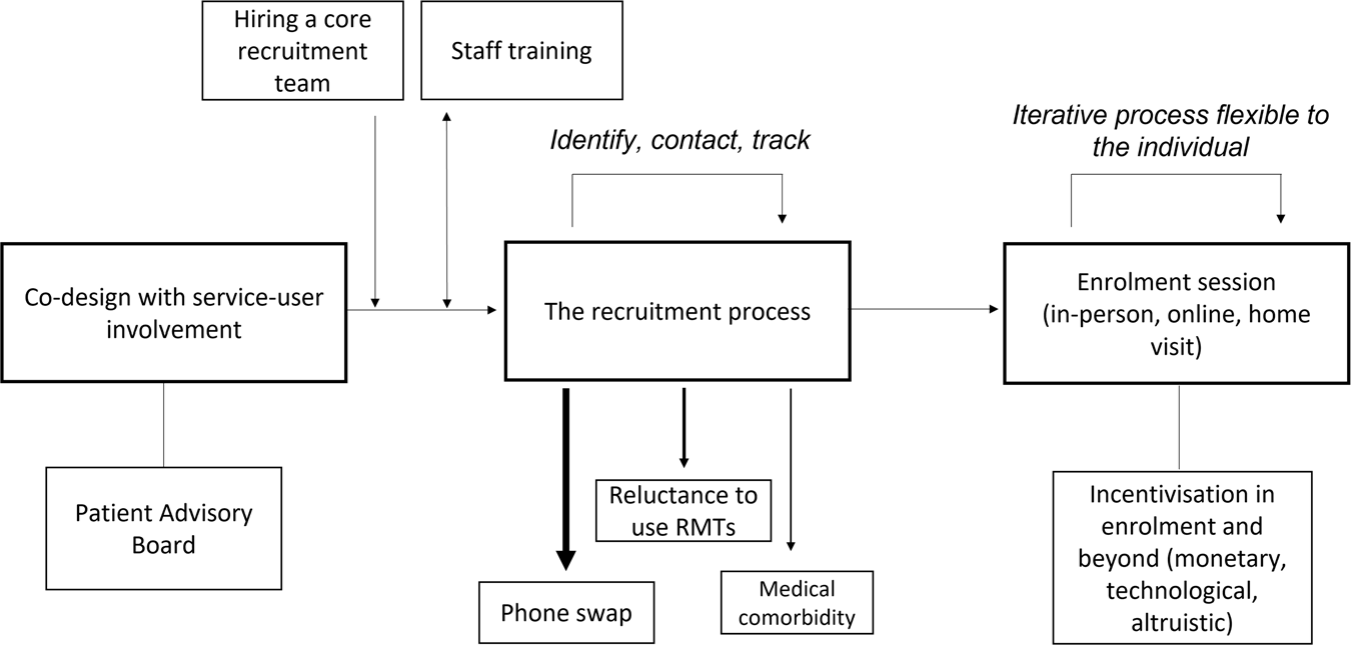
Lessons learned from recruitment into the RADAR-MDD project London site. Arrow weight for recruitment barriers indicates prevalence of this barrier, as seen in the RADAR-MDD project.

Figure 3 merges the reflections on strategies and barriers to participation from the RADAR-MDD study to depict the overall lessons learned during recruitment.

First, recruitment was preceded by thorough consultation with service users, and the acquisition of a suitably trained participant-facing team. Second, contacting eligible participants was streamlined, offering suitable financial and technological incentives during the recruitment and enrolment process. Third, we offered flexible enrolment procedures to suit individual needs and adapt to changing circumstances. While conducting this process, we learned that a consideration of barriers, such as phone swap, a reluctance to use the technologies, and medical comorbidities, will be key to maximising future recruitment.

## Discussion

This paper aimed to present the key lessons learned during recruitment into the RADAR-MDD London site, a longitudinal, multi-parametric RMT study. Despite varying reports of initial engagement in the previous literature^15,45^, in particular in MDD cohorts^19^, the study successfully recruited 350 participants over 31 months from the London centre, exceeding a preliminary target of 200. Following a recent focus on recruitment ‘lessons learned’ literature from the wider mHealth field, this paper mirrored this approach in the rapidly expanding RMT space. As such, we summarised our lessons learned into a model, covering reflections on recruitment strategies and obstacles to participating (Fig. 3).

### Implications for future study

This paper provides the foundations for understanding recruitment into multi-parametric RMT research. Future RMT studies should now build on the reflections presented here, alongside previous work, e.g., Druce et al.^31^, when considering their own recruitment design. Furthermore, we propose a need to create a framework of best practices that combines our insights with reflections from industry and other longitudinal studies (from different cultures, communities, populations) to better understand recruitment into RMT research. Such a body of work would provide for higher-quality trials with powered results to improve patient care.

Another valuable area of consideration for future work will be the extent to which successful recruitment into RMT studies is offset by feasibility. The RADAR-MDD study is part of the wider RADAR-CNS public-private partnership consortium, which involved a large amount of planning, resources, staff streamlining and funding before recruitment commenced. For the London site, this afforded three, full-time research assistants and access to service user groups. However, other projects might not have access to these resources, limiting the transferability of our reflections across studies. This reiterates the importance of future studies reflecting on their own lessons learned in recruitment, to build an inclusive framework for successful RMT recruitment that explores these trade-offs and their impacts on reaching recruitment targets.

In considering the COVID-19 climate, building upon the current work has never felt timelier. The RADAR-MDD study London site recruited between November 2017 and June 2020, and thus these results encapsulate both pre-pandemic and UK lockdown periods. Continuing to recruit during the pandemic has given the opportunity to exemplify RMTs as a viable and acceptable way to remotely collect health data. The transition towards a greater reliance on technology, as precipitated by COVID-19, may also act as a further facilitator of future RMT recruitment. Work should investigate the impact that the pandemic has had on recruitment strategies in various fields, and any resulting trends in utilising RMT in research and practice.

### Strengths and limitations

This paper reviews lessons learned solely from the London site of the RADAR-MDD study. It is important to acknowledge that where the fundamental recruitment and enrolment procedures remained largely similar across the remaining two sites in Barcelona and Amsterdam, several local and national processes impact on the translation of this model. For example, staff recruitment protocols in Amsterdam meant that staff were hired from a rotating pool of research assistants, rather than exclusively for the RADAR-MDD project. In Barcelona, the core recruitment team comprised a post-doctoral researcher and a part-time research assistant, with an additional 40 clinicians and health professionals promoting recruitment into the study. Also, differing ethical procedures across the three different countries led to discrepancies in incentivisation and payments offered to participants. This highlights the need for cross-cultural perspectives to be accounted for in future reflection work and any standardised framework for successful RMT recruitment.

It should also be noted that the recruitment strategies outlined in this paper do not include those which combat biases within the recruited sample. Reviews of recruitment into remote mHealth studies have found age, geographical and ethnical biases in samples^45^. The present study faced similar challenges. No concerted efforts to target recruitment towards these areas were conducted, due to the lack of current understanding of how best to conduct this in an RMT study. However, offering free technology could have reduced the risk of digital exclusion in our sample. Future work will focus on analysing participant demographics and provide more detail into potential biases in the sample.

A final limitation is the inability to analyse the demographics or technological abilities of those who refused participation. This would have allowed for a comparison with those who did participate, and an exploration of correlating factors. Further investigation might be possible for those recruited via C4C databases, for whom such data are retained. However, in the current study this would not be representative of the total participants contacted. Thus, future work building on these lessons learned should consider collecting this data as it could provide valuable insight into the type of individuals inclined to participate in an RMT trial.

Notwithstanding these limitations, the RADAR-MDD study is an exemplar of over-recruitment into an RMT study of MDD. This adds weight to recent work that suggests that participants with MDD advocate the use of technology to manage their health^12,13^, and provides an encouraging foundation for future work.

The current paper outlined key lessons learned for successful RMT recruitment in the RADAR-MDD project. These insights, alongside future researchers’ own reflections, should be used to build a model for successful recruitment into RMT research. These reflections will assist future researchers and stakeholders with a vested interest in the field. They will also promote transparency and encourage others to review their recruitment strategies, creating a more standardised, and successful, approach.

### Reporting summary

Further information on research design is available in the Nature Research Reporting Summary linked to this article.

## Supplementary information

Reporting Summary

## Data Availability

The data that support the findings of this study are available from the corresponding author upon reasonable request.
